# Prognostic prediction model construction and validation based on regional cerebral oxygen saturation and bispectral index monitoring in pediatric traumatic brain injury

**DOI:** 10.3389/fnins.2026.1866247

**Published:** 2026-10-16

**Authors:** Baowen Li, Ruhai Ma, Zhuyun Tian, Youcang Wan, Xiaoli Zhong, Wenhai Wang, Weixiao Yan, Shengkui Zhao

**Affiliations:** PICU, Qinghai Provincial Women and Children's Hospital, Xining, Qinghai, China

**Keywords:** bispectral index, nomogram, pediatric traumatic brain injury, prognosis, regional cerebral oxygen saturation

## Abstract

**Background:**

Continuous multimodal cerebral monitoring may improve detection and management of secondary injury after pediatric moderate-to-severe traumatic brain injury (msTBI). We developed and validated a prognostic model for 6-month functional outcome integrating regional cerebral oxygen saturation (rScO₂), bispectral index (BIS), clinical and biomarker data.

**Methods:**

Prospective cohort of 93 children with msTBI monitored with rScO₂ and BIS (monitoring group) and a retrospective control cohort of 66 children without such monitoring. Primary outcome was Glasgow Outcome Scale (GOS) at 6 months. Multivariate logistic regression identified independent predictors; a nomogram was constructed and internally validated using receiver operating characteristic (ROC), calibration curves, and decision curve analysis (DCA).

**Results:**

Total *N* = 159. Monitoring-group patients had shorter hospital and pediatric intensive care unit (PICU) lengths of stay and shorter invasive ventilation duration than controls (*p* < 0.05). Multivariate analysis identified admission Glasgow Coma Scale (GCS) [odds ratio (OR) 1.939, 95% confidence interval (CI): 1.261 ~ 2.984, *p* = 0.003], mean rScO₂ (OR 1.121, 95%CI: 1.027 ~ 1.225, *p* = 0.011), mean BIS (OR 1.115, 95%CI: 1.041 ~ 1.193, *p* = 0.002) and neuron-specific enolase (NSE) (OR 0.843, 95%CI: 0.769 ~ 0.924, *p* < 0.001) as independent outcome-associated variables. The nomogram achieved AUC 0.91 (95%CI: 0.84–0.97), with favorable calibration and net clinical benefit on DCA.

**Conclusion:**

A prognostic model combining admission GCS, mean rScO₂, mean BIS and NSE provides accurate individual risk estimation for pediatric msTBI and may assist bedside risk stratification and management decisions.

## Introduction

Traumatic brain injury (TBI) is a leading cause of mortality and disability among children worldwide, with an estimated global incidence of 50–60 million cases annually (Figaji, 2023). Moderate-to-severe traumatic brain injury (msTBI) accounts for a substantial proportion of pediatric TBI and remains a major clinical challenge due to its high mortality rate (15–30%) and long-term neurological sequelae (Figaji, 2023; Ben Abdeljelil et al., 2023; Chen et al., 2018). The pathophysiology of msTBI involves complex disturbances in cerebral hemodynamics, metabolism, and brain function, which can lead to cerebral ischemia and hypoxia, thereby aggravating secondary brain injury and directly affecting prognosis (Orr et al., 2024). Compared with adults, the management of pediatric TBI is associated with greater uncertainty because of developmental and physiological differences (Kochanek et al., 2019).

For decades, intracranial pressure (ICP) and cerebral perfusion pressure (CPP) monitoring have been considered standard components of TBI management. However, these parameters primarily reflect intracranial dynamics and provide only indirect information regarding cerebral oxygenation and metabolic status (Barud et al., 2021; Hawryluk et al., 2022). Consequently, cerebral tissue hypoxia or ischemia may not be detected in a timely manner using conventional monitoring alone.

Regional cerebral oxygen saturation (rScO₂) monitoring based on near-infrared spectroscopy (NIRS) enables continuous, real-time assessment of regional cerebral oxygenation and has been increasingly applied to evaluate cerebral perfusion and neurological recovery (Tsaousi et al., 2021). In parallel, the bispectral index (BIS), derived from electroencephalographic signals, provides dynamic information on cerebral functional activity and sedation depth. Previous studies have shown that BIS-guided sedation (target range 40–60) can significantly reduce the risk of intraoperative awareness under general anesthesia (Yan et al., 2018; Gomez et al., 2024).

Although rScO₂ and BIS monitoring have been progressively incorporated into adult TBI management, their application in pediatric TBI remains limited, and large-scale clinical studies are lacking (Mujawar et al., 2025; Godoy et al., 2020). Moreover, no established prognostic prediction model integrating rScO₂ and BIS parameters exists for children with TBI. Therefore, the present study aimed to evaluate the clinical value of rScO₂ and BIS monitoring in pediatric msTBI and to develop a prognostic prediction model incorporating these parameters with clinical variables, thereby improving early prognosis assessment, risk stratification, and therapeutic decision-making.

## Methods

### Study population and grouping

This study prospectively enrolled 93 children with msTBI who rScO_2_ and BIS monitoring at Qinghai Provincial Women and Children’s Hospital between December 2023 and December 2025 and were assigned to the monitoring group. In addition, 66 children with msTBI, treated between January 2021 and November 2023 without rScO₂ or BIS monitoring, were retrospectively reviewed and served as the control group. The inclusion criteria were as follows: (1) a definite history of traumatic brain injury and fulfillment of diagnostic criteria for moderate-to-severe TBI (Robinson, 2021); (2) GCS score between 3 and 12 (Araki et al., 2017); (3) hospital admission within 24 h after injury; (4) age ≤18 years. The exclusion criteria were as follows: (1) presence of diseases that could cause disturbances in water, electrolyte, or acid–base balance, such as diabetes mellitus, adrenal hyperfunction or hypofunction, other endocrine disorders, or inherited metabolic diseases; (2) neonatal hypoxic–ischemic encephalopathy; (3) hematological disorders.

This study was conducted in accordance with the Declaration of Helsinki and approved by the Ethics Committee of Qinghai Provincial Women and Children’s Hospital (Approval No: 2023KYLL-14). Informed consent was obtained from the legal guardians of all participating children.

### Management protocol

All patients were managed in accordance with the “Guidelines for the Management of Pediatric Severe Traumatic Brain Injury, Third Edition: Update of the Brain Trauma Foundation Guidelines” (Kochanek et al., 2019). Based on clinical and imaging assessments after admission, including blood pressure status and cranial computed tomography (CT) findings, emergency interventions such as blood pressure control and urgent surgical procedures were promptly implemented when indicated. Repeat cranial CT examinations were performed within 60 min whenever clinical deterioration occurred. All patients received standard comprehensive treatment, including maintenance of respiratory and circulatory stability, analgesia and sedation, intracranial pressure (ICP) reduction, correction of anemia, and follow-up cranial CT examinations or surgical interventions as clinically indicated.

### rScO_2_ monitoring

rScO_2_ Monitoring was monitored using a NIRS device and corresponding sensors manufactured by Masimo (USA). Patients were placed in the supine position, and the sensor was positioned at the midline of the forehead. In a resting state, cerebral oxygen saturation curves were continuously recorded for 3 min. Each patient underwent two measurements, and the mean value was used as the representative rScO_2_ level. Baseline rScO_2_ was measured within 1 h of hospital admission. To monitor changes in cerebral oxygenation, rScO_2_ was assessed every 6 h for a total duration of 7 days. This intermittent monitoring schedule was adopted as a practical approach based on our PICU’s routine assessment protocols and equipment availability, as well as to minimize the risk of sensor-related skin damage in pediatric patients. An absolute rScO_2_ value ≤50% or a decrease of ≥15% from baseline was defined as the warning threshold for cerebral oxygen supply–demand imbalance. Arterial blood gas analysis was simultaneously monitored to determine whether the rScO2 drop was driven by systemic hypoxemia. In cases of decreased arterial oxygen partial pressure (defined as PaO₂ < 60 mmHg), immediate measures to maintain airway patency were implemented, including timely suctioning, increasing the fraction of inspired oxygen, and adjusting ventilator parameters as needed, such as increasing positive end-expiratory pressure or modifying ventilation strategies to improve arterial oxygenation. Conversely, if the blood gas analysis was normal, clinical interventions were instead directed at optimizing local cerebral perfusion (e.g., adjusting blood pressure or managing intracranial pressure) in response to the isolated NIRS change. Following intervention, rScO_2_ was reassessed after 30–60 min to confirm improvements and determine further action.

### BIS monitoring

BIS monitoring was used to assist in the assessment of sedation depth and cerebral functional status. A bispectral index monitoring system and disposable electroencephalographic sensors (Covidien, USA) were employed. Disposable electrodes were placed on the midline of the forehead, above the right eyebrow, and at the right temporal region at the level of the lateral canthus. A signal quality index (SQI) > 50% and skin impedance <5kΩ were required to ensure reliable signal acquisition. The BIS system recorded values every hour for 7 consecutive days, with automatic recording to ensure continuous data collection. During the early phase after admission (within the first 72 h), a BIS range of 40–60 was used as the target for sedation management based on previous studies (Yan et al., 2018). Persistent BIS values >60 accompanied by agitation, a trend toward elevated ICP, or marked stress responses indicated inadequate sedation, prompting adjustment of sedative and analgesic regimens. Conversely, persistent BIS values <40 or the emergence of burst suppression patterns suggested excessive sedation or cerebral functional suppression, leading to appropriate reduction of sedative dosages. After the initial 72 h, as the peak risk of acute intracranial hypertension subsided and spontaneous cortical activity naturally recovered, maintaining a strict BIS target below 60 became clinically inappropriate and difficult to achieve without excessive medication. Therefore, the sedation strategy was transitioned to an individualized approach. Sedative doses were progressively reduced according to the patient’s clinical trajectory to avoid iatrogenic over-sedation, facilitate daily neurological assessments, and support the awakening process.

### Data collection and outcome measures

The observational variables included age, sex, body weight, mechanism of injury (traffic accident, falls from height, ground-level falls, or Striking/Blunt), type of brain injury (subdural hematoma, epidural hematoma or, subarachnoid hemorrhage, cerebral contusion or diffuse axonal injury, mixed intracranial injuries involving two or more types), time from injury to hospital admission, surgical intervention, GCS score at admission, PRISM III score at admission, oxygenation index at admission, mean arterial pressure at admission, NSE level at admission, cerebrospinal fluid (CSF) lactate level at admission, CSF albumin level at admission, GOS score at 6 months after injury, 28-day mortality after admission, total hospital length of stay, PICU length of stay, duration of invasive Mechanical Ventilation, mean rScO_2_ values after admission, and mean BIS values after admission.

Prognosis was assessed 6 months after injury using the GOS through telephone or outpatient follow-up. Clinical prognosis were compared between the two groups. All follow-up assessments were conducted by a PICU physician who was blinded to group allocation. According to GOS at 6 months after injury, patients in the monitoring group were further classified into a good prognosis group (GOS ≥ 4, *n* = 5 0) and a poor prognosis group (GOS ≤ 3, *n* = 43).

Furthermore, to evaluate the clinical management associated with multimodal monitoring, we retrospectively extracted detailed data regarding specific neuromonitoring alert events (defined as rScO₂ ≤ 50% or a ≥ 15% decrease; BIS > 60 or <40/burst suppression). For these instances, we recorded the specific clinical and pharmacological interventions deployed. To quantify the physiological responses to these interventions, pre-intervention and post-intervention (30–60 min) rScO₂ and BIS values were extracted for comparison. For NIRS alert events, temporally matched arterial blood gas results (specifically PaO₂) were also collected to assess their correlation with rScO₂. Finally, the overall completeness of the scheduled 7-day neuromonitoring data was systematically audited, accounting for missed sessions due to off-unit clinical procedures or pre-mature mortality.

### Statistical analysis

Continuous variables were presented as median (interquartile range, IQR) and compared using the Mann–Whitney *U* test. Categorical variables were compared using the *χ*^2^ test or Fisher’s exact test, as appropriate. Missing data for core neuromonitoring and clinical variables were addressed using complete case analysis, and patients with incomplete records were excluded from the final model development. Variables with *p* < 0.05 in univariate analysis entered into multivariate logistic regression; PRISM III excluded due to collinearity. A nomogram was built in R and internally validated with 1,000 bootstraps; performance assessed by AUC, calibration, and DCA. Additionally, sensitivity analyses were conducted by evaluating the model’s performance exclusively in survivors and in the subgroup of patients with severe TBI (GCS 3–8). A secondary analysis was also performed using 28-day mortality as the primary predictive outcome to further validate the model’s utility. A *p* value <0.05 was considered statistically significant.

## Results

### Comparison of clinical characteristics between monitoring group and control group

A total of 159 children with msTBI were included in this study, comprising 79 males and 80 females. The median age was 5.1 (2.2, 8.3) years, and the mean body weight was 19.8 (13.6, 27.9) kg. Comparisons between the monitoring group and the control group revealed no significant differences in age, sex, mechanism of injury, type of brain injury, time from injury to admission, surgery, GCS score, PRISM III score, oxygenation index at admission, mean arterial pressure at admission, NSE, CSF lactate, and CSF albumin (all *p* > 0.05; Table 1). Notably, all 159 patients (100%) in this cohort, including the 18 patients classified with moderate TBI, were intubated and required invasive mechanical ventilation. Furthermore, a careful review of the medical records confirmed that none of the enrolled patients suffered from abusive head trauma (AHT).

**Table 1:** Comparison of clinical data between two groups of pediatric msTBI patients.

Parameters	Monitoring group (*n* = 93)	Control group (*n* = 66)	*Z*/*χ*^2^	*p*
Age (years)	5.0 (2.3,7.9)	6.8 (2.2,9.9)	−1.516	0.130
Male, *n* (%)	45 (48.4)	34 (51.5)	0.151	0.697
Weight (kg)	19.0 (14.0,25.0)	20.0 (12.0,34.2)	−0.810	0.419
Mechanism of injury				
Traffic accident, *n* (%)	44 (47.3)	36 (54.5)	0.808	0.369
Falls from height, *n* (%)	32 (34.4)	19 (28.8)	0.560	0.454
Ground-level falls, *n* (%)	12 (12.9)	8 (12.1)	0.021	0.884
Striking/Blunt, *n* (%)	5 (5.7)	3 (4.5)	0.000	1.000
Type of brain injury			5.547	0.236
Subdural hematoma, *n* (%)	12 (12.9)	7 (10.6)		
Epidural hematoma, *n* (%)	17 (18.3)	11 (16.7)		
Subarachnoid hemorrhage, *n* (%)	14 (15.1)	5 (7.6)		
Cerebral contusion or diffuse axonal injury, *n* (%)	11 (11.8)	16 (24.2)		
Mixed intracranial injuries (≥2 types), *n* (%)	39 (41.9)	27 (40.9)		
Injury-to-admission time >4 h, *n* (%)	24 (25.8)	22 (33.3)	1.064	0.302
Time from admission to biomarker collection (h)	0.7(0.5,0.8)	0.7(0.6,0.8)	−1.035	0.301
Surgery, *n* (%)	59 (63.4)	39 (59.1)	0.309	0.578
GCS score at admission	6.0(5.0,7.5)	6.0(5.0,8.0)	−0.600	0.548
TBI severity classification				
Severe TBI (GCS 3–8), *n* (%)	86 (92.5)	55 (83.3)	3.170	0.075
Moderate TBI (GCS 9–12), *n* (%)	7 (7.5)	11 (16.7)		
PRISM III score at Admission	13.0(9.0,15.0)	13.5(7.0,20.0)	−0.538	0.591
Oxygenation index at admission	257.0(227.5,279.0)	249.5(186.0,333.0)	−0.697	0.486
Mean arterial pressure at admission (mmHg)	72.0(67.0,80.5)	75.5(71.0,80.3)	−1.952	0.051
NSE (ng/ml)	35.2(27.8,40.4)	34.1(28.9,40.5)	−0.474	0.636
CSF albumin (g/L)	2.5(2.1,2.9)	2.6(2.1,3.2)	−1.143	0.253
CSF lactate (mmol/L)	3.6(3.1,4.2)	3.5(3.0,4.0)	−1.017	0.309
Invasive mechanical ventilation, *n* (%)	93(100.0)	66(100.0)	-	-
ICP monitoring, *n* (%)	60(64.5)	41(62.1)	0.160	0.689
Decompressive craniectomy, *n* (%)	26(28.0)	16(24.2)	0.274	0.601

Data are presented as median (interquartile range, IQR) or *n* (%); The patients were divided into a monitoring group and a control group based on whether rScO_2_ and BIS monitoring were performed; msTBI, moderate to severe traumatic brain injury; GCS, Glasgow Coma Scale; PRISM III score, pediatric risk of mortality III; NSE, neuron-specific enolase; CSF, cerebrospinal fluid.

### Comparison of clinical prognosis between monitoring group and control group

There is not statistically significance in 28-day mortality rate between monitoring group and the control group (*p* > 0.05). Among the total 159 patients, 34 died during hospitalization, with a median time from admission to death of 5.0 (4.0, 6.0) days. Specifically, 16 patients in the monitoring group died before completing the planned 7-day neuromonitoring. In contrast, the total hospital length of stay, PICU length of stay, and duration of invasive mechanical ventilation were significantly shorter in the monitoring group compared with the control group (all *p* < 0.05). In addition, the GOS score at 6 months after injury was significantly higher in the monitoring group than in the control group (*p* < 0.05) (Table 2).

**Table 2:** Comparison of clinical outcomes between two groups of pediatric msTBI patients.

Group	28-day mortality (%)	Total length of stay (d)	The length of PICU stay (d)	Duration of invasive mechanical ventilation (d)	GOS score at 6 months
Multimodal monitoring treatment group (*n* = 93)	18 (19.4)	28.0 (24.5, 33.0)	18.0 (13.5, 20.0)	9.0 (5.0, 12.0)	4.0 (3.0, 5.0)
Routine monitoring treatment group (*n* = 66)	21 (31.8)	41.0 (7.0, 44.0)	23.0 (7.0, 28.0)	12.0 (5.0, 15.0)	4.0 (1.0, 4.0)
*χ^2^/Z*	3.239	−4.559	−3.338	−2.076	−2.603
*p*	0.072	<0.001	0.001	0.038	0.009

Data are presented as median (interquartile range, IQR) or *n* (%); the patients were divided into a monitoring group and a control group based on whether rScO2 and BIS monitoring were performed; msTBI, moderate to severe traumatic brain injury; GOS, Glasgow Outcome Scale.

### Clinical interventions and neuromonitoring signal responses

During the study period, 81 patients in the monitoring group experienced a total of 424 NIRS alert events (defined as absolute rScO₂ ≤ 50% or a decrease of ≥ 15% from baseline). Among these, 329 events (77.6%) had temporally matched arterial blood gas results available, while the remaining 95 instances lacked concurrent data and were treated as missing. Of the 329 events with matched data, the partial pressure of arterial oxygen (PaO₂) was < 60 mmHg in 109 instances (33.1%) and ≥ 60 mmHg in 220 instances (66.9%). Spearman correlation analysis revealed a moderate positive correlation between rScO₂ and PaO₂ during these alert events (*r* = 0.428, *p* < 0.001). The clinical interventions implemented in response included airway suctioning (*n* = 204, 48.1%), increasing fraction of inspired oxygen (FiO₂) (*n* = 120, 28.3%), adjusting positive end-expiratory pressure (PEEP) (*n* = 131, 30.9%), fluid resuscitation (*n* = 317, 74.8%), adjusting vasoactive drugs (*n* = 328, 77.4%), head elevation (*n* = 162, 38.2%), and mannitol administration (*n* = 323, 76.2%). Following these interventions, the median rScO₂ significantly increased from 42.0% (37.0%, 45.0%) pre-intervention to 45.0% (41.0%, 50.0%) at 30–60 min post-intervention (*Z* = −9.436, *p* < 0.001).

Regarding BIS monitoring during the first 72 h of admission, we recorded 124 clinically significant BIS alert events in 71 patients. For the 75 events with elevated BIS (>60), subsequent strengthening of sedation and/or analgesia (implemented in 74 instances, 98.7%) significantly decreased the median BIS from 67.0 (64.0, 73.0) to 63.0 (58.0, 68.0) (*Z* = −5.214, *p* < 0.001). Conversely, for the 49 events presenting with persistent BIS < 40 and/or burst suppression patterns, reducing or temporarily interrupting sedation (implemented in 43 instances, 87.8%) resulted in a significant increase in the median BIS from 35.0 (33.0, 36.0) to 38.0 (35.0, 41.0) (*Z* = −4.190, *p* < 0.001).

### Completeness of neuromonitoring data

During the 7-day monitoring period, brief interruptions occurred when patients were temporarily transferred out of the unit for imaging, surgery, or other clinical procedures. For patients who died before completing the 7-day period, monitoring data were only included up to the time of death, and subsequent scheduled time points were not classified as missing data. Consequently, for NIRS, out of 2,448 planned intermittent monitoring sessions, 2,203 valid data points were recorded, yielding a completion rate of 90.0%, with 245 sessions (10.0%) missed due to off-unit clinical activities. For continuous BIS monitoring, out of 14,688 planned hourly data points, 13,099 valid data points were acquired (completion rate: 89.2%), while 1,589 data points (10.8%) were missed for similar clinical reasons.

### Logistic regression analysis for predicting prognosis in children with msTBI

Univariate analysis demonstrated that significant differences existed between the two groups in GCS score, PRISM III score, mean rScO_2_ values, mean BIS values, NSE, CSF lactate levels and mean arterial pressure at admission (all *p* < 0.05; Supplementary Table 1). These factors were included in the multivariate logistic regression analysis. To mitigate the potential impact of multicollinearity, the PRISM III score was excluded from the model. The admission GCS score (OR = 1.821, 95%CI: 1.174 ~ 2.824, *p* = 0.007), mean rScO_2_ values (OR = 1.111, 95%*CI*: 1.016 ~ 1.215, *p* = 0.021), mean BIS values (OR = 1.113, 95%*CI*: 1.039 ~ 1.193, *p* = 0.002), and NSE (OR = 0.843, 95%*CI*: 0.767 ~ 0.926, *p* < 0.001) were independent predictors of poor prognostic in children with msTBI (Supplementary Table 2).

### Development and evaluation of the prognostic prediction model for pediatric msTBI

A nomogram-based prognostic prediction model was constructed based on the four independent predictors identified by multivariate logistic regression analysis, including admission GCS score, mean rScO_2_ values, mean BIS values, and NSE. Each variable was assigned a weighted score according to its relative contribution, and the total score corresponded to the predicted probability of poor prognostic, enabling individualized risk estimation (Figure 1).

**Figure 1:**
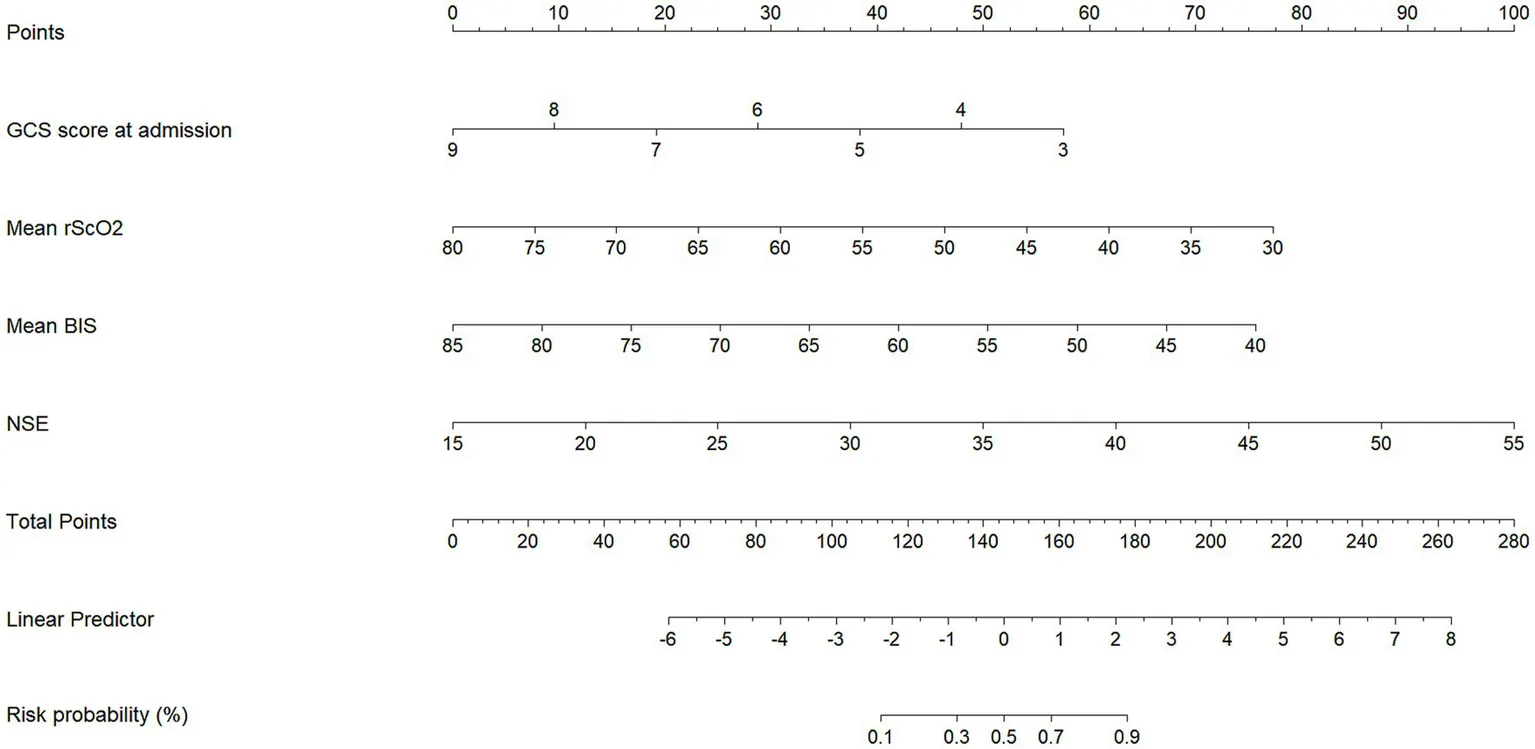
Nomogram of the prognostic prediction model for pediatric patients with msTBI. msTBI, moderate to severe traumatic brain injury; rScO2, regional cerebral saturation oxygenation; BIS, bispectral index; GCS, Glasgow Coma Scale; NSE, neuron-specific enolase.

The area under the ROC curve (AUC) of the model was 0.91 (95%CI: 0.84 ~ 0.97) (Figure 2). The sensitivity and specificity of the predicted probability were 76.7 and 92.0%, respectively. The *C*-index for predicting poor prognostic in children with msTBI was 0.91, indicating excellent discriminative ability of the model. The calibration curve showed strong agreement between the nomogram-predicted and observed clinical outcomes (Figure 3). Furthermore, decision curve analysis (DCA) demonstrated that the model provided substantial net clinical benefit across a threshold probability range of 10–95%, highlighting its potential clinical utility in predicting poor prognosis in children with msTBI (Supplementary Figure 1).

**Figure 2:**
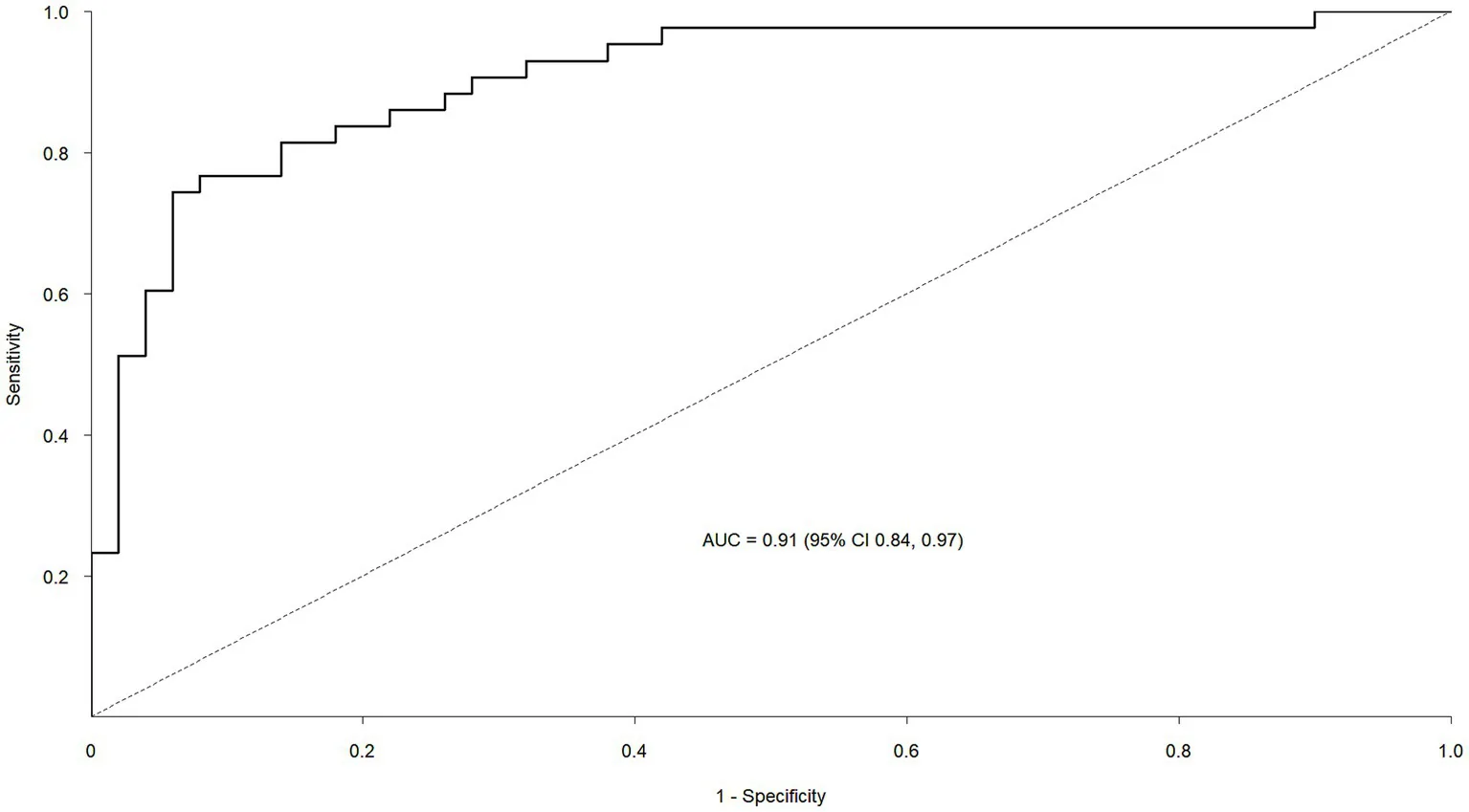
ROC curve of the nomogram model for predicting the prognosis in pediatric patients with msTBI. msTBI stands for moderate to severe traumatic brain injury, ROC represents receiver operating characteristic, and AUC denotes area under the curve.

**Figure 3:**
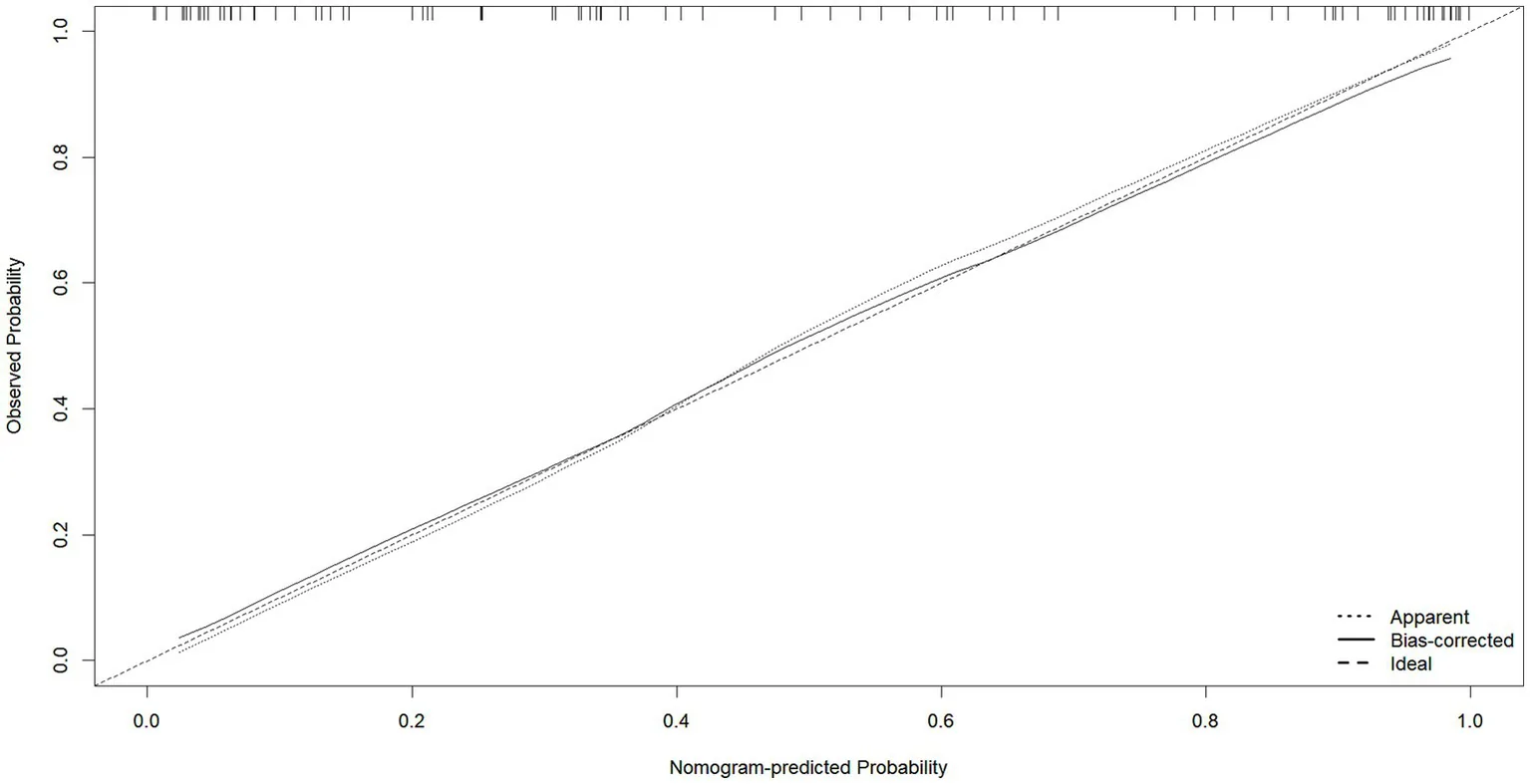
Calibration curve of the nomogram model for predicting the prognosis in pediatric patients with msTBI. msTBI stands for moderate to severe traumatic brain injury.

### Sensitivity and secondary analyses

To ensure the robustness of our prognostic model and confirm that its performance was not disproportionately biased by the non-survivor cohort (28-day mortality: 19.4%), we conducted a series of sensitivity analyses. When restricting the analysis exclusively to the 75 survivors in the monitoring group, the model maintained high discriminative ability for predicting a poor 6-month functional outcome (GOS = 3), yielding an AUC of 0.876. Furthermore, a sensitivity analysis performed strictly on the severe TBI subgroup (GCS 3–8; *n* = 86) similarly demonstrated excellent predictive performance with an AUC of 0.900. Finally, in a secondary analysis utilizing 28-day mortality as the primary predicted outcome, the combined model (incorporating GCS, mean rScO₂, mean BIS, and NSE) achieved an AUC of 0.901, indicating strong utility in predicting early mortality alongside long-term functional deficits. Spearman correlation was used to assess the relationship between rScO₂ and PaO₂ during NIRS alert events. A *p* value <0.05 was considered statistically significant.

### Incremental predictive value of multimodal monitoring

To quantify the added prognostic value of the neuromonitoring tools and biomarkers over standard clinical assessment, we compared the performance of multiple nested models. The baseline clinical model utilizing admission GCS alone demonstrated moderate predictive ability for poor functional outcome (AUC = 0.744). The addition of the biomarker NSE to GCS improved the predictive accuracy (AUC = 0.813). Similarly, combining GCS with the neuromonitoring variables (mean rScO₂ and mean BIS) resulted in a substantial increase in performance (AUC = 0.836). Ultimately, the comprehensive model integrating clinical assessment, multimodal neuromonitoring, and NSE (GCS + mean rScO₂ + mean BIS + NSE) achieved the highest discriminative ability (AUC = 0.907). This confirms that the incorporation of continuous rScO₂ and BIS monitoring, alongside NSE, provides significant incremental value in prognosticating pediatric msTBI. Furthermore, the longitudinal trends of daily rScO₂ and BIS values over the 7-day monitoring period for the good and poor prognosis groups are detailed in Supplementary Figure 2.

## Discussion

This study systematically evaluated the clinical value of combined rScO₂ and BIS monitoring in pediatric msTBI. Previous studies have lacked large-scale validation of these monitoring techniques in pediatric patients and have not developed integrated prognostic models that incorporate both physiological and neurophysiological parameters (Donnelly et al., 2017). This study fills that gap by demonstrating that the implementation of combined rScO₂ and BIS monitoring was associated with better in-hospital outcomes and long-term neurological prognosis in children with msTBI. Specifically, our results show that this combined monitoring approach correlated with reduced hospital and ICU length of stay, as well as a shorter duration of mechanical ventilation. While these associations suggest potential benefits in managing secondary brain injury, the historical control design precludes definitive causal conclusions, which would require future randomized controlled trials.

Furthermore, the prognostic model incorporating multimodal cerebral monitoring parameters and biomarkers exhibited good discrimination, calibration, and net clinical benefit, indicating its potential utility for risk stratification and prognosis prediction in pediatric msTBI.

Secondary brain injury following msTBI represents a critical determinant of poor prognosis. Its core pathophysiological mechanisms include inadequate cerebral perfusion, cerebral tissue hypoxia, metabolic imbalance, and abnormalities in neuronal electrical activity (Robinson, 2021). In pediatric msTBI, these pathophysiological changes are more complex due to the unique characteristics of the developing brain, including immature cerebral autoregulation (Araki et al., 2017; Donnelly et al., 2017). This immaturity makes the pediatric brain more vulnerable to fluctuations in cerebral oxygenation and metabolic balance, thereby enhancing the predictive value of rScO_2_ and BIS in this population. As such, a single monitoring modality is frequently insufficient for a comprehensive assessment. The growing emphasis on multimodal monitoring (MMM) and individualized therapy underscores the need for techniques that offer real-time, comprehensive insights into cerebral health (Makarenko et al., 2016). rScO_2_, based on near-infrared spectroscopy, allows continuous assessment of local cerebral oxygenation and serves as an important indicator for evaluating cerebral perfusion and oxygen metabolic balance (Roldán and Kyriacou, 2021). The BIS, as an integrated parameter reflecting the degree of electroencephalographic suppression, provides indirect assessment of cerebral functional integrity and neuroregulatory status (Vinciguerra and Bösel, 2017). Previous studies have shown that mean rScO_2_ values in patients with TBI are significantly lower than those in healthy controls (Yang et al., 2022). In a study by Senapathi et al. (2017), involving 78 patients with TBI, mean BIS values differed significantly among patients with mild, moderate, and severe head injuries, and BIS values were positively correlated with GCS scores, suggesting that BIS may serve as a useful adjunct for assessing levels of consciousness and neurological function. However, existing evidence has largely focused on adult TBI populations or perioperative monitoring settings, whereas data regarding pediatric msTBI remain relatively limited.

In the present study, rScO_2_ and BIS were jointly applied for continuous monitoring in children with msTBI, and necessary interventions-including airway suctioning, adjustment of ventilator parameters, and optimization of analgesia and sedation regimens—were implemented according to monitoring results. The lack of significant reduction in 28-day mortality, despite improvements in long-term outcomes, can be explained by the focus of rScO_2_ and BIS monitoring on reversible secondary brain injuries, such as ischemia, hypoxia, and metabolic imbalances. These monitoring tools help detect and manage these issues early, improving recovery and reducing hospital stay. However, fatal primary injuries, like severe brain damage or hemorrhage, cannot be reversed by monitoring and continue to impact mortality rates. In a pediatric TBI study, near-infrared spectroscopy–based rScO_2_ monitoring was shown to capture cerebral oxygenation changes associated with neurological events, such as seizures, in a subset of patients (Lang et al., 2024). Furthermore, a review focusing on TBI highlighted that NIRS has been widely used for the assessment of cerebral oxygenation and cerebrovascular autoregulation/reactivity, supporting its role as a noninvasive bedside monitoring tool in the dynamic management of secondary brain injury (Roldán and Kyriacou, 2021). On the other hand, studies have also reported that during barbiturate therapy in patients with TBI, BIS monitoring can reflect drug-induced burst-suppression electroencephalographic patterns and shows good correlation with conventional electroencephalography (EEG) findings (Cottenceau et al., 2012). It is important to note that while BIS is traditionally utilized to gauge sedation depth, in msTBI, lower BIS values often reflect severe intrinsic cortical depression or primary structural brain injury rather than merely pharmacological effects. In this context, BIS-guided intervention did not mandate the inappropriate reduction of Tier 1 neuroprotective sedation. Instead, an unexpected or disproportionate drop in BIS served as an early warning trigger for urgent diagnostic re-evaluation—such as ICP assessment or neuroimaging—to rule out impending secondary insults. Furthermore, BIS monitoring optimized management by preventing unnecessary over-sedation, thereby avoiding iatrogenic hypotension and ensuring the maintenance of adequate cerebral perfusion pressure. The results of the present study further suggest that continuous combined monitoring with rScO_2_ and BIS, coupled with targeted interventions, may facilitate early identification and correction of reversible secondary injury factors.

Identifying independent prognostic predictors and developing a prediction model for pediatric msTBI are of important clinical relevance in light of the potential value of multimodal monitoring. The admission GCS score, mean rScO_2_ values, mean BIS values, and NSE remained independent predictors of poor prognosis. The GCS score, as a core clinical indicator reflecting the degree of neurological impairment at admission, has been widely used for severity stratification and prognostic evaluation in pediatric TBI. Previous studies have demonstrated that lower GCS scores are significantly associated with increased mortality risk and unfavorable neurological prognosis (Zuniga et al., 2024). Neuron-specific enolase, as a biomarker of neuronal injury, exhibited an odds ratio less than 1, indicating that elevated NSE levels were significantly associated with poor prognosis. This finding is consistent with previous reports (Cheng et al., 2014) and further supports the clinical value of NSE in prognostic assessment of pediatric TBI.

The prognostic prediction model developed in this study for pediatric msTBI demonstrated good predictive performance. The model achieved an area under the receiver operating characteristic curve (AUC) of 0.91 (95%CI: 0.84 ~ 0.97) and a *C*-index of 0.91, indicating strong discriminative ability. Calibration curve analysis showed close agreement between the predicted probabilities and observed outcomes, suggesting good model stability. Our model integrates a multimodal approach, including neurological function assessment, cerebral oxygenation, electroencephalographic activity, and biomarkers. This comprehensive integration of variables provides a more holistic view of the patient’s condition, better reflecting the multifactorial pathophysiology of pediatric msTBI. Compared with single scoring systems or single-parameter predictors, the present model integrates neurological function assessment, cerebral oxygen metabolism monitoring, and biomarker information, which better reflects the multifactorial and multimechanistic pathophysiology of pediatric msTBI.

Several limitations of this study should be acknowledged. First, as highlighted earlier, the use of an observational design with a historical control cohort precludes any definitive causal conclusions regarding whether combined monitoring directly improves outcomes; specifically, because the monitoring and control cohorts were treated during different time periods (2023–2025 versus 2021–2023, respectively), we cannot completely rule out historical control bias. Potential improvements in standard care, evolving clinical experience, or other changes in the healthcare system over time may have favorably influenced the outcomes independently of the monitoring itself. Therefore, future randomized controlled trials are essential to establish true causality. Second, the interpretation of BIS in this population is complex; because lower BIS values may fundamentally reflect the intrinsic severity of the primary structural brain injury rather than solely the pharmacological depth of sedation, isolating the effect of sedation management from baseline neurological status remains challenging. Third, the reliance on intermittent rScO₂ assessments (every 6 h) rather than continuous monitoring is a limitation, as it may have precluded the detection of transient cerebral hypoxic episodes. Finally, this was a single-center study with a relatively limited sample size, which may introduce selection bias and affect the power of the study, particularly in performing subgroup analyses where the ability to detect differences across various patient subsets (e.g., age, injury type, or severity) was constrained. Consequently, validation through a multicenter collaborative study and confirmation of the model’s robustness in a larger cohort are highly desirable. Incorporating additional monitoring techniques like cerebral blood flow assessment, neuro-metabolic imaging, and ICP monitoring could improve the model’s accuracy. Exploring its applicability across different age groups and severity levels will assess its robustness. Moreover, the application of time-series analysis and artificial intelligence–based algorithms may further enhance the accuracy and clinical applicability of prognostic prediction for pediatric msTBI.

## Conclusion

Combined rScO₂ and BIS monitoring is associated with improved clinical trajectories and facilitates the detection of pathophysiological changes in brain perfusion and metabolism in pediatric msTBI. A nomogram combining admission GCS, mean rScO₂, mean BIS and NSE accurately estimates the risk of poor outcome and may assist bedside decision making. However, future randomized controlled trials comparing monitoring-guided treatment versus standard of care are required to determine whether this approach directly optimizes clinical management.

## Data Availability

The original contributions presented in the study are included in the article/Supplementary material, further inquiries can be directed to the corresponding authors.

## Supplementary material

SUPPLEMENTARY FIGURE 1 Decision curve analysis of the nomogram model for evaluating poor prognosis in pediatric patients with msTBI. msTBI stands for moderate to severe traumatic brain injury.

SUPPLEMENTARY FIGURE 2 Longitudinal trends of daily rScO2 and BIS values in the good and poor prognosis groups. **(A)** Daily median rScO2 values over the 7-day monitoring period. **(B)** Daily median BIS values over the 7-day monitoring period. Data are presented as daily median (interquartile range). For patients who died during the 7-day monitoring period, only pre-mortem monitoring data were included.
