# Supplementary material for: Prognostic prediction model construction and validation based on regional cerebral oxygen saturation and bispectral index monitoring in pediatric traumatic brain injury
Source: Front Neurosci. 2026 Oct 16;20:1866247. doi: 10.3389/fnins.2026.1866247 (PMC13719534; doi:10.3389/fnins.2026.1866247)
Supplement: Supplementary file 1 [file Table_1.DOCX]

**Supplementary Table1 Univariate analysis of prognostic-related indicators in the msTBI pediatric monitoring group.**

| Parameters | Good prognosis group (n=50) | Poor prognosis group (n=43) | Z/χ2 | *p* |
| --- | --- | --- | --- | --- |
| Age (years) | 4.0 (2.0,7.5) | 5.9 (3.2,8.0) | -1.357 | 0.176 |
| Male, n (%) | 24 (48.0) | 21 (48.8) | 0.006 | 0.936 |
| Weight (kg) | 16.8 (12.2,23.5) | 22.0 (16.7,27.9) | -1.938 | 0.053 |
| Mechanism of injury |  |  |  |  |
| Traffic accident, n (%) | 20 (40.0) | 24 (55.8) | 2.319 | 0.128 |
| Falls from height, n (%) | 19 (38.0) | 13 (30.2) | 0.618 | 0.432 |
| Ground-level falls, n (%) | 8 (16.0)) | 4 (9.3) | 0.923 | 0.337 |
| Striking/Blunt, n (%) | 3 (6.0) | 2 (4.7) | 0.000 | 1.000 |
| Type of brain injury |  |  | 1.131 | 0.889 |
| Subdural hematoma, n (%) | 7 (14.0) | 5 (11.6) |  |  |
| Epidural hematoma, n (%) | 9 (18.0) | 8 (18.6) |  |  |
| Subarachnoid hemorrhage, n (%) | 9 (18.0) | 5 (11.6) |  |  |
| Cerebral contusion or diffuse axonal injury, n (%) | 6 (12.0) | 5 (11.6) |  |  |
| Mixed intracranial injuries (≥2 types), n (%) | 19 (38.0) | 20 (46.5) |  |  |
| Injury-to-admission time >4 h, n (%) | 14 (28.0) | 10 (23.3) | 0.272 | 0.602 |
| Surgery, n (%) | 33 (66.0) | 26 (60.5) | 0.305 | 0.581 |
| GCS score at Admission | 7.0 (5.8,8.0) | 5.0 (5.0,6.0) | -4.128 | ＜0.001 |
| PRISM III score at Admission | 10.0(8.0,13.0) | 15.0 (13.0,18.0) | -5.224 | ＜0.001 |
| Oxygenation index at admission | 262.5 (240.8,286.5) | 254.0 (205.0,275.0) | -1.649 | 0.099 |
| Mean arterial pressure at admission (mmHg) | 75.5 (69.0,81.0) | 69.0 (64.0,79.0) | -2.194 | 0.028 |
| Mean rScO₂ | 65.0 (61.0,68.0) | 62.0 (55.0,67.0) | -2.385 | 0.017 |
| Mean BIS | 66.0 (58.8,73.3) | 57.0 (50.0,65.0) | -4.018 | ＜0.001 |
| NSE (ng/ml) | 32.1 (27.7,35.7) | 39.6 (35.2,46.8) | -4.196 | ＜0.001 |
| CSF albumin (g/L) | 2.5 (2.1,2.8) | 2.6 (2.1,3.0) | -1.316 | 0.188 |
| CSF lactate level at admission (mmol/L) | 3.4 (2.9,3.9) | 3.9 (3.4,4.3) | -2.592 | 0.010 |

Data are presented as median (interquartile range, IQR) or n (%); msTBI，moderate to severe traumatic brain injury；rScO2，regional cerebral saturation oxygenation；BIS，Bispectral index；GCS，Glasgow Coma Scale；PRISM III score，Pediatric risk of mortality Ⅲ；NSE，Neuron-Specific Enolase；CSF，Cerebrospinal fluid；

**Supplementary Table2 Multivariate Logistic stepwise regression analysis for predicting the prognosis of children with msTBI.**

| Observation index | β | SE | Wald | OR | 95%CI | *p* |
| --- | --- | --- | --- | --- | --- | --- |
| GCS score at admission | 0.599 | 0.224 | 7.173 | 1.821 | (1.174~2.824) | 0.007 |
| Mean arterial pressure at admission | 0.034 | 0.031 | 1.224 | 1.034 | (0.974~1.099) | 0.269 |
| Mean rScO_2_ | 0.105 | 0.046 | 5.343 | 1.111 | (1.016~1.215) | 0.021 |
| Mean BIS | 0.107 | 0.035 | 9.159 | 1.113 | (1.039~1.193) | 0.002 |
| NSE | -0.171 | 0.048 | 12.714 | 0.843 | (0.767~0.926) | ＜0.001 |
| CSF lactate level at admission | -0.610 | 0.389 | 2.465 | 0.543 | (0.254~1.164) | 0.116 |

msTBI，moderate to severe traumatic brain injury；rScO_2_，regional cerebral saturation oxygenation；BIS，Bispectral index；GCS，Glasgow Coma Scale；NSE，Neuron-Specific Enolase；CSF，Cerebrospinal fluid；
